# 

**Published:** 1890-08-09

**Authors:** 


SATURDAY, AUGUST 9th, 1890.

**Tested by Results** - 273

**Words of Consolation.**—God's Hand - 274

**The Doctor's Pulpit.**—Bright's Disease and Life Insurance 275

**Life in a London Hospital.** II. - 276

**Before the Lords' Committee.** X.— Last of this Session 277

**August** - 278

**Everybody's Page** - 279

**Notes and News** - 280

**Annotations.**—Cleanliness and Health — 'Rubbishin'—Hypnotism and the Daily Press - 282

**Thoughts on Ill-Health.** I.—Not Quite "F.t" - 285

**The Practitioner's Mirror and Retrospect:**—

MEDICINE—The Treatment of Diabetes—Venesection, 283; Injections of Ether in Neuralgia - 284

SURGERY—Restoration of Bone after Trephining—Cure of Cancer by Erysipelas - 284

THERAPEUTICS—Salol, 284; Poisoning by Antifebrin—Lanolin—Prevention of the Toxic Effects of Cocaine - 285

**The Student's Bookshelf.**—Anatomy - 287

**General Charities** - 287

**Passing Topics**—"The Lancet" and Hospital Nurses - 288

**Scraps and Gleanings** - 288

EXTRA SUPPLEMENT—THE NURSING MIRROR.

**En Passant**—The London Hospital—A Russian Story—Massage—Notes from America—How She Got Off—Portsmouth District Nurses—The Spirit of Discontent - lxxix
**Sketches of Insanity.**—By Francis H. Walmsley, M.D. VI.—Melancholia - lxxx
**Dr. Tarrant's Mistake.**—(*Continued*) - lxxx
**A Note from Portugal** - lxxxi
**Everybody's Opinion.**—Workhouse Nurses, lxxxi; An American Note—For or Against? - lxxxii
**Notes and Queries** - lxxxii
**Appointments—Vacancies** - lxxxii
**Amusements and Relaxation** - lxxxii

